# Identification of disease-related genes in *Plasmodium berghei* by network module analysis

**DOI:** 10.1186/s12866-023-03019-0

**Published:** 2023-09-21

**Authors:** Junhao Lin, Shan Zeng, Qiong Chen, Guanghui Liu, Suyue Pan, Xuewu Liu

**Affiliations:** Department of Neurology, Nanfang Hospital, Southern Medical University, Guangzhou, 510515 China

**Keywords:** *Plasmodium berghei*, Network module, Experimental cerebral malaria (ECM), Pathogenesis

## Abstract

**Background:**

*Plasmodium berghei* has been used as a preferred model for studying human malaria, but only a limited number of disease-associated genes of *P. berghei* have been reported to date. Identification of new disease-related genes as many as possible will provide a landscape for better understanding the pathogenesis of *P. berghei*.

**Methods:**

Network module analysis method was developed and applied to identify disease-related genes in *P. berghei* genome. Sequence feature identification, gene ontology annotation, and T-cell epitope analysis were performed on these genes to illustrate their functions in the pathogenesis of *P. berghei*.

**Results:**

33,314 genes were classified into 4,693 clusters. 4,127 genes shared by six malaria parasites were identified and are involved in many aspects of biological processes. Most of the known essential genes belong to shared genes. A total of 63 clusters consisting of 405 *P. berghei* genes were enriched in rodent malaria parasites. These genes participate in various stages of parasites such as liver stage development and immune evasion. Combination of these genes might be responsible for *P. berghei* infecting mice. Comparing with *P. chabaudi*, none of the clusters were specific to *P. berghei*. *P. berghei* lacks some proteins belonging to *P. chabaudi* and possesses some specific T-cell epitopes binding by class-I MHC, which might together contribute to the occurrence of experimental cerebral malaria (ECM).

**Conclusions:**

We successfully identified disease-associated *P. berghei* genes by network module analysis. These results will deepen understanding of the pathogenesis of *P. berghei* and provide candidate parasite genes for further ECM investigation.

**Supplementary Information:**

The online version contains supplementary material available at 10.1186/s12866-023-03019-0.

## Introduction

Malaria is one of the three major infectious diseases in the world. According to the World Health Organization report, approximately 627,000 people worldwide died from malaria in 2020 [1]. Severe malaria is the leading cause of death [2]. To illustrate the pathogenesis of malaria parasite, rodent malaria parasite is commonly used as a disease model. Currently, at least four *Plasmodium* species are known to infect mice, namely, *Plasmodium berghei*, *Plasmodium yoelii*, *Plasmodium chabaudi*, and *Plasmodium vinckei* [3]. Rodent *Plasmodium* species are used in different disease models to investigate different aspects of human malaria given the differences in their biological properties. For example, *P. berghei* is usually used as a model of severe malaria to study the pathogenesis of cerebral malaria and maternal malaria [4]; *P. chabaudi* is utilized to clarify the drug resistance and immune evasion [5]; and *P. yoelii* is employed to elucidate the biology of hepatic stages of *Plasmodium* species and the mechanism of natural and acquired immunity against liver stages [6]. *P. berghei* is easily maintained in laboratory mice, and recombinant DNA technology has been widely used to manipulate the parasite genome [7]. Thus, this species has become the preferred model for studying *Plasmodium* gene functions and parasite–host interactions efficiently. To date, the number of disease-related genes reported in *P. berghei* is limited. The discovery of disease-causing genes in *P. berghei* will facilitate further understanding of the pathogenesis of human malaria and adoption of appropriate strategies to fight against malaria infections. However, more than 5,000 protein-coding genes are present in *P. berghei* genome [3, 8], and identifying disease-related genes as many as possible is challenging.

The development of high-throughput sequencing technology and the widespread use of bioinformatics have facilitated the discovery of disease-related genes through comparison of *Plasmodium* genomes with different phenotypes [9]. For example, we previously found that two enzymes involve in vitamin B1 synthesis and metabolism are present in human malaria parasites but absent in rodent malaria parasites by comparing the genomes of human malaria parasites to rodent malaria parasites [10]. It is consistent with the previous report that vitamin B1 deprivation reduces the proliferation rate of *P. falciparum* [11]. Through the comparison of genomes between artemisinin-resistant and sensitive *P. falciparum*, Ariey et al. found that mutations in *PF3D7_1343700*, which encodes a putative Kelch protein, is responsible for artemisinin resistance [12]. In a previous study, we identified several malaria-related genes by comparative analysis of protein-coding genes from *P. falciparum* with those from *P. knowlesi* and *P. vivax*. Further experimental studies revealed that the *P. falciparum*-specific protein: parasite-infected erythrocyte specific protein 2 (PIESP2) is involved in the adhesion of infected erythrocytes to brain microvascular endothelial cells [13]. However, most of the current studies prefer to identify disease-related genes in human malaria parasites and lack the comprehensive exploration of the genome of model *Plasmodium* parasites such as *P. berghei*. To better understand the mechanism of pathogenesis of *P. berghei*, the disease-related genes of *P. berghei* genome need to be identified through comparisons of the *Plasmodium* genomes with different phenotypes or biological properties.

In this study, we developed a network modularity approach to identify the disease-related genes in *P. berghei* genome. We compared all protein sequences of three human and three rodent *Plasmodium* species to construct a large, disconnected network based on sequence alignment. Then, the network was partitioned into multiple network modules or clusters using the network module analysis method BGLL [14]. A network module refers to a cluster where node connections within the same cluster are denser than those from different clusters [15]. Proteins within the same network module were similar in their sequences and close in their functions. The number of protein sequences from six *Plasmodium* species in each module or cluster were calculated, which resulted in the enrichment of each cluster in six species. Genes shared by all *Plasmodium* species, genes specific to or under expansion in the rodent malaria parasites, and genes possibly contributing to the occurrence of cerebral malaria in *P. berghei* genome could be identified by analyzing the enrichment of the abovementioned clusters in six *Plasmodium* species. This study not only provides a basis for better understanding of the pathogenesis of *P. berghei* in mice but also presents a direction for experimentally illustrating the molecular mechanism of human cerebral malaria using *P. berghei* as a model malaria parasite.

## Materials and methods

### Protein sequence acquisition and sequence alignment

Protein sequences of three rodent *Plasmodium* species including *P. berghei* ANKA, *P. chabaudi*, and *P. yoelii* 17XNL and three human *Plasmodium* species including *P. falciparum* 3D7, *P. knowlesi* strain H, and *P. vivax* Sal-1 were obtained from the PlasmoDB database (https://plasmodb.org) [16]. After sequences with length less than 50 a.a. were removed, we obtained 5069 sequences from *P. berghei* ANKA, 5211 sequences from *P. chabaudi*, 6601 sequences from *P. yoelii* 17XNL, 5532 sequences from *P. falciparum* 3D7, 5320 sequences from *P. knowlesi* strain H, and 5580 sequences from *P. vivax* Sal-1. These sequences were combined, and pairwise sequence alignment was implemented using HMMER 3.3.1 [17]. An alignment with E-value ≤ 1E-10 was considered to be significant. In the end, a binary matrix $A={\left[{a}_{ij}\right]}_{33,314\times 33,314}$ which represents sequence similarity was obtained, where ${a}_{ij}$ stands for the alignment between protein *i* and *j*. If protein *i* and *j* hit mutually in alignment analysis, then ${a}_{ij}$=1; otherwise, ${a}_{ij}=0$. The obtained matrix can be converted into a large network.

### Network analysis by BGLL algorithm

The obtained large network is disconnected and consists of thousands of independent components. For each component, the BGLL algorithm was applied for network module or cluster discovery. BGLL is a fast modularity optimization method. It contains two steps. First, each node belongs to its own cluster. A cluster was formed by moving a node into the group of neighborhood nodes when the modularity gain is positive and maximum. The modularity gain is computed by.

$$\Delta Q = \left[ {\frac{{{\sum _{in}} + 2{k_{i,in}}}}{{2m}} - {{\left( {\frac{{{\sum _{tot}} + {k_i}}}{{2m}}} \right)}^2}} \right] - \left[ {\frac{{{\sum _{in}}}}{{2m}} - {{\left( {\frac{{{\sum _{tot}}}}{{2m}}} \right)}^2} - {{\left( {\frac{{{k_i}}}{{2m}}} \right)}^2}} \right]$$

where ${\sum }_{in}$ is the sum of weight of edges within the cluster, ${\sum }_{tot}$ is the sum of weight of edges linked to cluster, ${k}_{i}$ is the sum of weights incident to the cluster, ${k}_{i,in}$is the sum of weights of edges from node *i* to the cluster, and m represents the number of edges in the network. This process is applied for all nodes until the value of modularity no longer improves. In the second step, a new network is built by considering the clusters found in the first step as nodes. The weight of the edge between two nodes is calculated by sum of weights of the edges in the clusters. The two steps are repeated until no improvement can be achieved or the threshold of modularity is achieved. Several thresholds ranging from 0.1 to 0.6 were tested.

In this study, the BGLL algorithm was modified for disconnected network. We first used the graph shortest path algorithm (*Dijkstra* algorithm) to identify each independent component. Then, for each component, BGLL algorithm was applied for module recognition through the depth-first search (DFS) strategy [18]. By this method, 33,314 protein sequences were partitioned into thousands of network modules or clusters. The number of proteins from six *Plasmodium* species in each cluster was calculated to obtain the enrichment of all clusters in each malaria parasite. The program was implemented in MATLAB 2020a.

### Sequence feature identification, T-cell epitope prediction, and gene ontology annotation

We utilized DeepTMHMM (https://dtu.biolib.com/DeepTMHMM) with default parameters to identify signal peptide and transmembrane domain in a protein sequence [19]. T-cell epitopes in a protein sequence were analyzed through the webserver NetMHCpan EL4.1 of the Immune Epitope Database (https://www.iedb.org/home_v3.php) [20, 21]. Epitopes with percentile rank ≤ 0.01 were kept. Gene ontology (GO) annotation was performed via the webserver of the PlasmoDB database. A gene set with *p* ≤ 0.05 was considered to be statistically significant.

## Results

### Protein sequence alignment, network construction, and cluster analysis

**Fig. 1:**
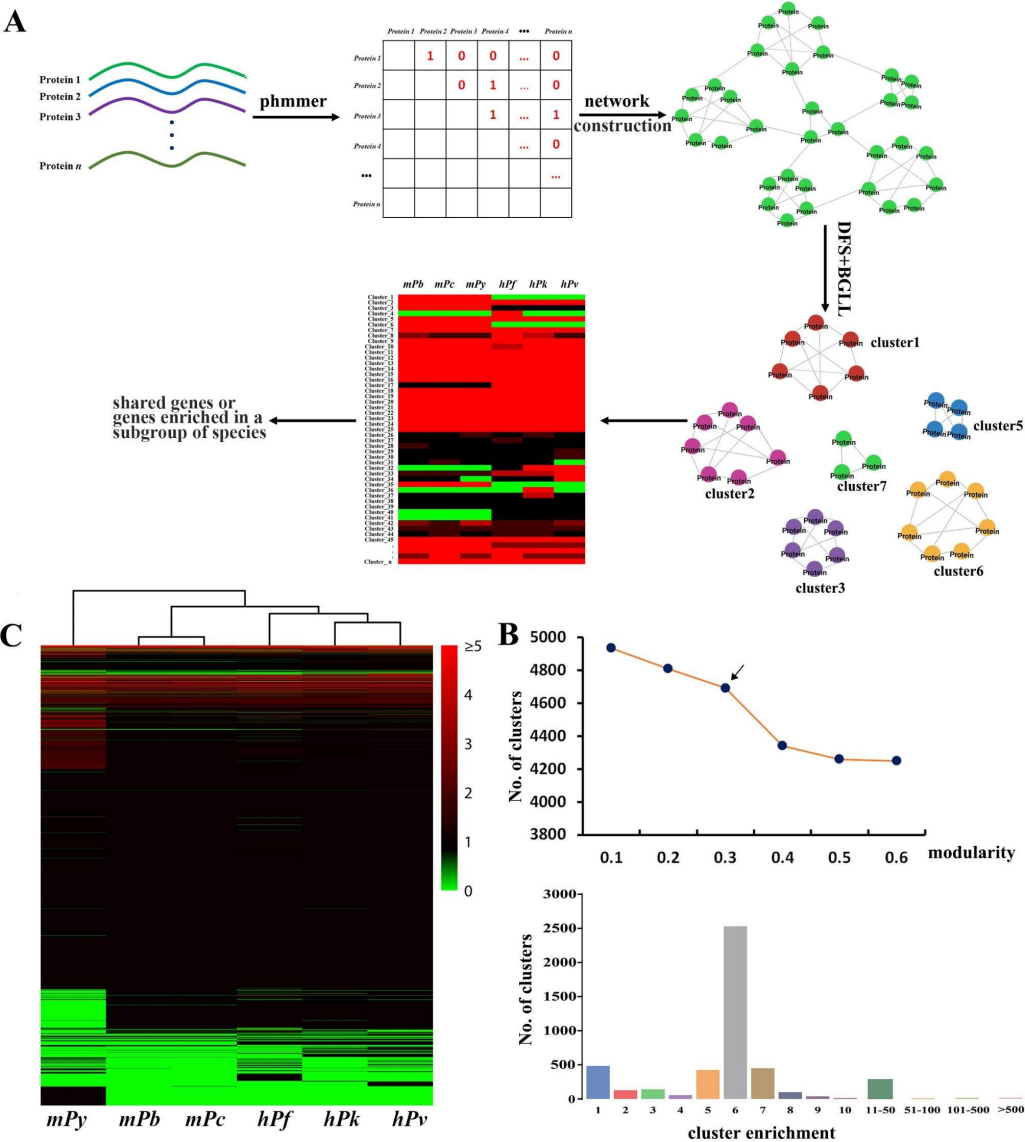
Network module analysis of protein sequences from six *Plasmodium* species. **(A)** Workflow of network analysis. Sequence alignment was performed using phmmer, which resulted in a matrix representing sequence similarity. The matrix was converted into a large, disconnected network. Graph shortest path was introduced to identify all the components of the network and the BGLL algorithm was adopted to discover the modules within each component by DFS strategy. The number of proteins from six *Plasmodium* species in each module or cluster was calculated, which resulted in the enrichment of each cluster in six species. **(B)** Upper panel shows the number of network modules at different thresholds identified by BGLL. Arrow indicates the modularity chosen in this study. Lower panel shows the number of clusters at different enrichment. **(C)** Heatmap showing the clusters in six *Plasmodium* species. Hierarchical clustering analysis of six species based on the enrichment of 4693 clusters. Green, black, and red indicate cluster values equal to zero, one, and higher than one, respectively

As shown in Fig. 1A, 33, 314 protein sequences collected from three rodent and three human malaria parasites underwent pairwise alignment using phmmer with an expectation value of E-10, which resulted in 8,801,145 hits. A protein sequence similarity matrix $A={\left[{a}_{ij}\right]}_{33,314\times 33,314}$ was constructed, where ${a}_{ij}=1$ represents protein *i* and *j* mutually hit; otherwise, ${a}_{ij}=0$. This matrix was a sparse upper triangular matrix and can be transformed into a large network with 33,314 nodes. The network was disconnected and consisted of 4,235 independent components. The BGLL algorithm, which is a fast greedy method to identify the clusters or modules within the large network, was applied because of the huge number of nodes. A module was defined that the number of edges within the modules was significantly higher than that of edges among modules. BGLL is modified for identification of clusters within connected network. Several modularity values were tested (Fig. 1B, upper panel). Notably, the number of clusters decreased when the cut-off value was increased from 0.3 to 0.4. Meanwhile, modularity values between 0.3 and 0.7 usually indicate the existence of a strong modular structure [22]. Therefore, we set the threshold value to 0.3. In the end, 33,314 protein sequences were classified into 4693 clusters (Supplementary Table 1).

The number of protein sequences from six *Plasmodium* species in each cluster was calculated, which resulted in the enrichment of each cluster in six malaria parasites. A large number of clusters contained six proteins from different *Plasmodium* species (Fig. 1B, lower panel). A total of 484 clusters comprised only one node, which implied that these clusters were specific to a particular *Plasmodium* species. Cluster1 was the largest cluster among these clusters. It contained 1096 proteins, 174 of which were from *P. berghei*. All proteins in Cluster1 were specific to the rodent malaria parasites but absent in human malaria parasites. Hierarchical clustering analysis of six *Plasmodium* species based on the enrichment of all clusters suggested that *P. berghei* and *P. chauchadi* were closely related while *P. yoelii* was distantly related to five other *Plasmodium* species (Fig. 1C). By comparing the enrichment of each cluster in *Plasmodium* species with different properties, we can identify genes shared by all *Plasmodium* species, genes unique to or under expansion in rodent malaria parasites, and genes probably contributed to experimental cerebral malaria in *P. berghei*.

### Identification of genes shared by six *Plasmodium* species

**Fig. 2:**
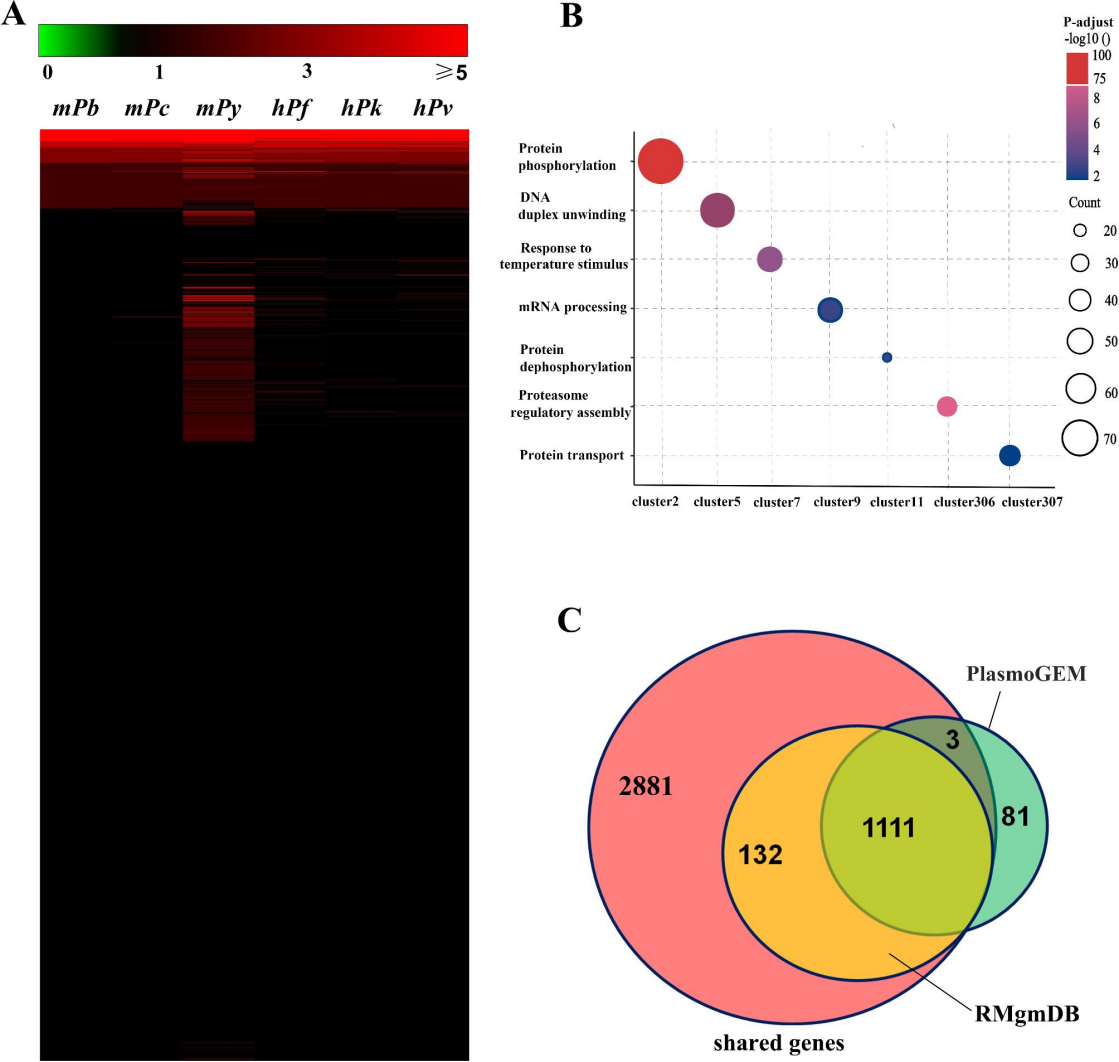
Identification of genes shared by *Plasmodium* species. **(A)** Heatmap of the enrichment of 3,367 clusters shared by six *Plasmodium* species. Green, black, and red indicate cluster values of each *Plasmodium* species equal to zero, one, and higher than five, respectively. **(B)** Functional annotation of clusters with enrichment ≥ 15 in *P. berghei*. The color shade was scaled to adjust *p* value of the most significantly enriched gene set. Circle size represents the number of proteins in clusters. **(C)** Venn diagram showing the overlap between shared genes (red circle) and essential genes derived from RMgmDB (yellow circle) or PlasmoGEM (green circle) database

*P. berghei* shares many biological properties with other *Plasmodium* species, such as possessing a similar life cycle and being capable of parasitizing erythrocytes [23]. Thus, we speculated that the presence of some genes shared by all malaria parasites might be responsible for such biological processes. The maximum and minimum values of each cluster were calculated to identify such genes in *P. berghei* genome. For a given cluster, it is considered to be a shared cluster when its minimum value is greater than 0 and maximum value is less than 5 times the minimum value. By this method, we finally obtained 3,367 clusters containing 4,127 *P. berghei* genes (Supplementary Tables 2 and 3), most of which contained one protein-encoding gene from each of the six *Plasmodium* species (Fig. 2A, black area).

The clusters with enrichment ≥ 15 in *P. berghei* underwent functional analysis (Fig. 2B). Cluster2 contained the largest number of proteins, 71 of which are from *P. berghei*. Proteins in this cluster are protein kinases, including serine/threonine and tyrosine protein kinases. These proteins are involved in many biological processes, such as intracellular signaling, protein phosphorylation, cell motility regulation, and cell cycle regulation. Cluster5 consisted of 36 *P. berghei* proteins. Most of them are RNA unwinding enzymes which participate in DNA duplication and gene transcriptional regulation. Cluster306 contained ATPase and proteasome subunits which are involved in sexual production and proteasome assembly regulation. Cluster7 comprised members from the DNAJ protein family which are involved in protein folding and response to temperature stimulus. Cluster11 mainly contained phosphatases responsible for protein dephosphorylation.

Bushell et al. identified 1,195 essential genes by knocking out 2,575 *P. berghei* genes [24]. Disruption of the essential genes leads to significant growth inhibition or apoptosis of *P. berghei*. Meanwhile, the Rodent Malaria genetically modified DataBase (RMgmDB) contains information on genetically modified malaria parasites generated by labs worldwide [25]. This database comprises 1243 essential genes. We found through analysis that 1114 essential genes from the Plasmodium Genetic Modification Project (PlasmoGEM) database were included in the shared genes. All 1243 genes from RMgmDB were also included in the shared genes (Fig. 2C). These results indicate that most of the essential genes are shared by six *Plasmodium* species. Therefore, identification of the shared genes is not only for facilitating the discovery of new essential genes in *P. berghei* and improving the understanding of the pathogenesis of all six parasites but also for providing new possible targets to fight against malaria infection.

### Identification of genes enriched in rodent malaria parasites

In general, *Plasmodium* infection is host specific. For example, the rodent malaria parasite *P. berghei* is only capable of infecting mice, while *P. falciparum* is a human *Plasmodium*. Though *P. falciparum* can invade mouse erythrocytes, this parasite is unable to develop after invasion. We compared the enrichment of each cluster in rodent malaria parasites with that in human malaria parasites to identify genes in *P. berghei* required for infecting mice. A cluster was considered to be enriched in the rodent malaria parasites when its minimum value in rodent malaria parasite was more than 5-fold higher than its maximum value in human *Plasmodium* species.

**Fig. 3:**
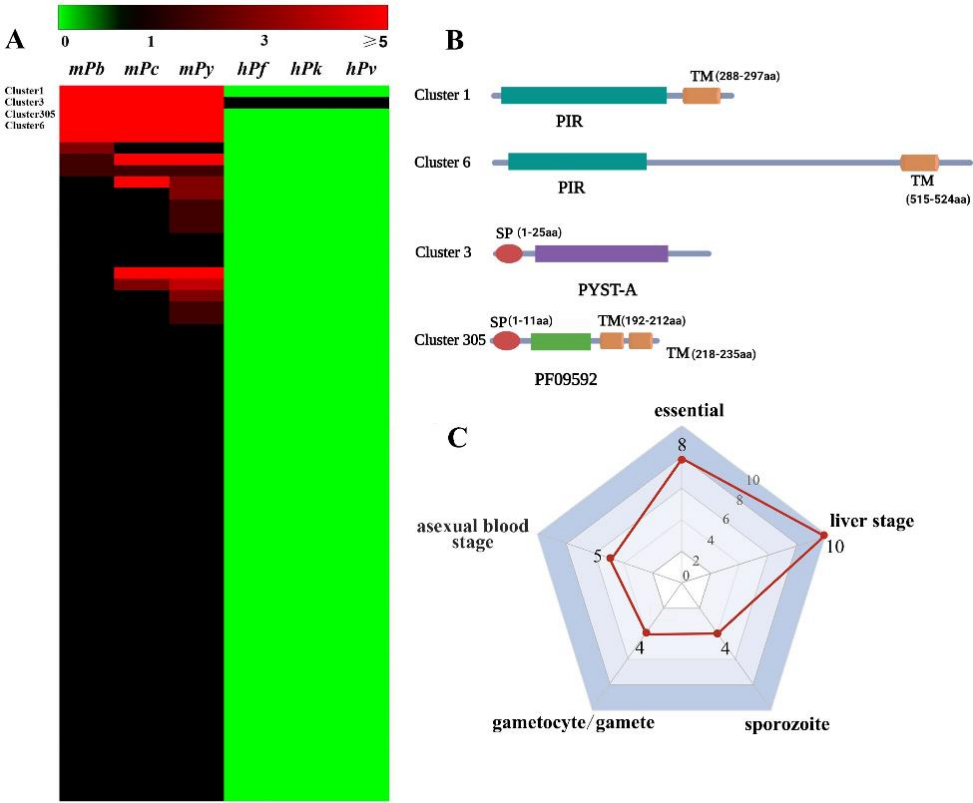
Identification of genes contributing to infect mice in *P. berghei*. **(A)** Heatmap showing the enrichment of 63 clusters enriched in rodent malaria parasites. Cluster1, Cluser3, Cluster305, and Cluster6 are the top four clusters with cluster value ≥ 40 in *P. berghei.* Green, black, and red indicate cluster values equal to zero, one, and higher than five, respectively. **(B)** Domain architectures of proteins from the top four clusters. SP, signal peptide. TM, transmembrane domain. PIR, Plasmodium Interspersed Repeat. **(C)** Radar plot illustrating the phenotypic manifestation of 30 rodent *Plasmodium* specific genes after disruption obtained from RMgmDB.

As shown in Fig. 3A, we identified 63 clusters containing 405 *P. berghei* proteins (Supplementary Table 4). A total of 62 clusters were unique to the rodent malaria parasites, and one cluster, Cluster3, expanded in the rodent malaria parasites. Cluster1, Cluster3, Cluster305, and Cluster6 were the top four clusters enriched in rodent malaria parasites. Sequence feature analysis of proteins from Cluster1 and Cluster6 showed that all members contain Plasmodium Interspersed Repeat (PIR) domains (Fig. 3B), and most of them possess signal peptides and transmembrane domains and are displayed on the surface of infected erythrocytes. The PIR domain contains eight α-helices and a long loop [26]. The loop is located on the surface of the protein structure, and its length and amino acid composition varies significantly among different members, which allowed *Plasmodium* to escape from the host immune attack. Cluster1 and Cluster6 members contained PIR structural domains, but they differed in protein length (about 300 a.a. in Cluster1 and about 600 a.a. in Cluster6). As a result, they were classified into two independent network modules. Cluster3 contained 75 *P. berghei* proteins with unknown functions. This cluster was present in all six *Plasmodium* species but was expanded in rodent malaria parasites, particularly in *P. yoelii*. We found through sequence analysis that approximately half of proteins (36/74, 48.6%) contained signal peptides and only two proteins contained transmembrane domains, which suggested that most of these proteins would be exported. Cluster305 contained proteins with unknown functions. Members in this cluster contained a signal peptide and two transmembrane domains (Fig. 3B). Proteins with such feature are usually located in Maurer’s cleft [27]. All these proteins with unknown functions need further investigation.

Functionally analysis of 68 *P. berghei* proteins from the remaining 59 clusters revealed that many of these proteins had unknown functions (Supplementary Table 5). Only a few proteins had been investigated. For example, PBANKA_0100600 is a schizont membrane-associated cytoadherence protein. It assists infected erythrocytes to adhere to the vascular endothelium in a CD36-dependent manner, subsequently avoiding being cleared by the spleen [28]. PBANKA_0501200 is upregulated in infective sporozoites gene 4 (UIS4) protein, which is highly expressed in the infective sporozoite stage and developing liver stage. Disruption of this gene does not affect sporozoite invasion of hepatocytes or transformation into liver early liver stage. However, parasites lacking UIS4 are severely impaired in development of early liver stage and thus in their ability to establish infection of host [29]. Several proteins were annotated as exported proteins which might be transported from inner parasitophorous vacuoles to cytoplasm or extracellular of infected erythrocytes. Although most rodent *Plasmodium* specific genes have unknown functions, the phenotype information of 30 of them after depletion can be obtained from RMgmDB. As shown in Fig. 3C, the phenotypic manifestation of these genes after disruption covers various stages of *Plasmodium* life cycle. A total of 10 genes are required for liver stage development of parasites [24, 28–30]. Depletion of these genes leads to an apparently reduced transition from liver stage to blood stage. And 8 genes are necessary for parasite growth in blood stage [24, 31–33]. In conclusion, the rodent plasmodium-specific proteins participate in various stages of *P. berghei* and combination of these genes might be responsible for overall ability of *P. berghei* to infect mice.

### Identification of genes related to experimental cerebral malaria in *P. berghei*

**Fig. 4:**
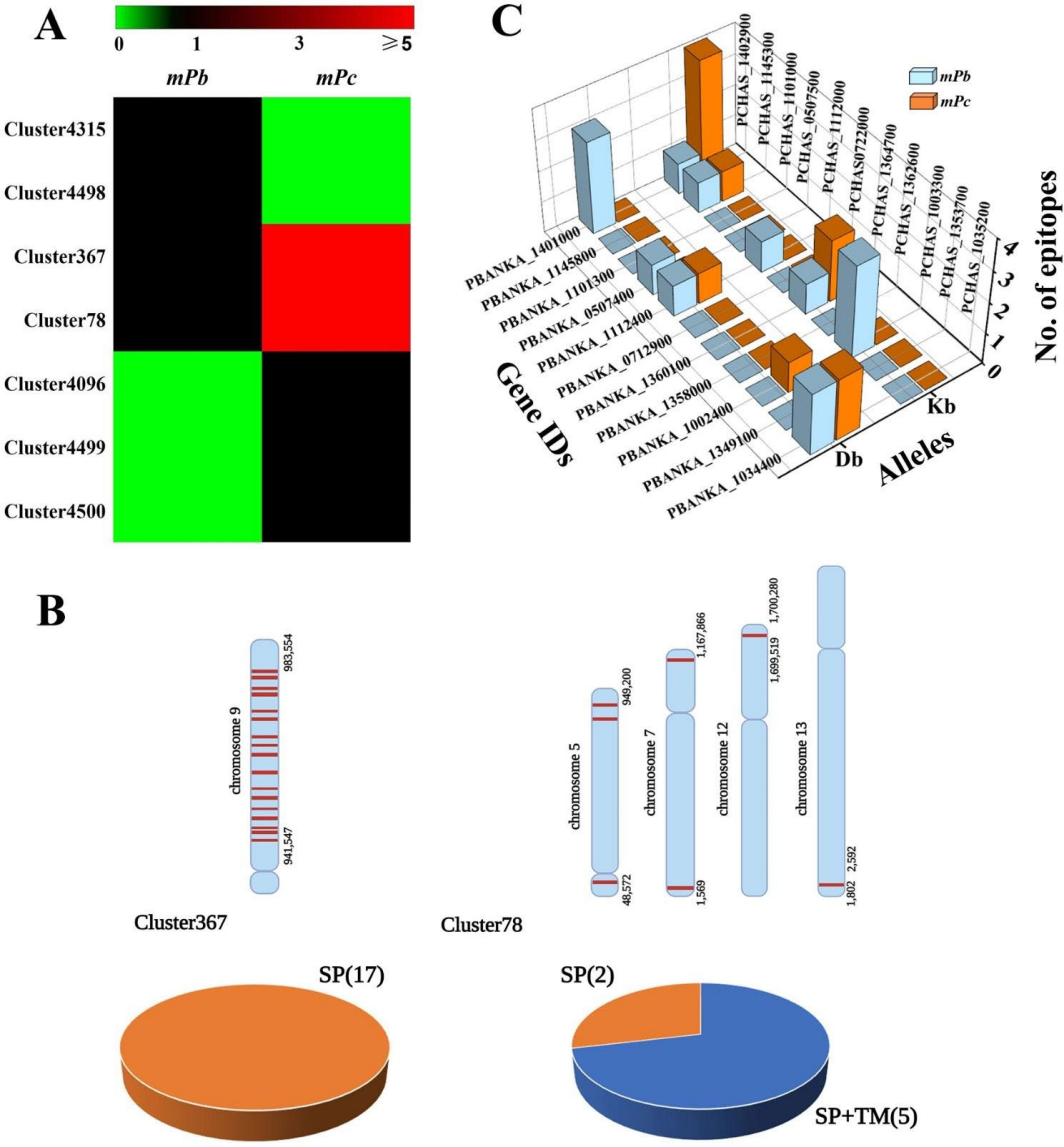
Identification of genes involved in the development of ECM. **(A)** Heatmap showing the enrichment of 2 and 5 clusters enriched in *P. berghei* and *P. chabaudi*, respectively. Green, black, and red indicate cluster values equal to zero, one, and higher than five, respectively. **(B)** Left panel, the chromosomal locations of 17 protein-coding genes in Cluster367 and sequence features of their protein products. Right panel, the chromosomal locations of 7 protein-coding genes in Cluster78 and sequence features of their protein products. SP, signal peptide. TM, transmembrane domain. **(C)** The number of predicted epitopes of 11 ECM-related proteins in *P. berghei* and their orthologs in *P. chabaudi* when class-I MHC alleles are *Db* and *Kb*

Although there are at least four rodent *Plasmodium* species, only *P. berghei* infection of C57BL/6 mice can lead to the development of experimental cerebral malaria (ECM) in mouse [34]. Therefore, the presence or absence of certain proteins in *P. berghei* might be responsible for the occurrence of ECM. To identify the proteins in *P. berghei* associated with the development of ECM, we compared the enrichment of each cluster in *P. berghei* with that in *P. chabaudi* and identified two and five clusters enriched in *P. berghei* and *P. chabaudi*, respectively (Fig. 4A and Supplementary Table 6).

All genes contained in the clusters unique to *P. berghei* were pseudogenes, which suggested that no clusters were enriched in *P. berghei*. Among *P. chabaudi*-enriched clusters, Cluster367 and Cluster78 were expanded in *P. chabaudi*. After 4 pseudogenes in Cluster367 were removed, the remaining 17 protein-coding genes in Cluster367 were clustered in the region of Chr9: 941,547–983,554 (Fig. 4B, left panel). All these proteins contained signal peptides (Fig. 4B, left panel), which implied that they might be exported proteins. Cluster78 contained 7 protein-coding genes distributing on four different chromosomes (Fig. 4B, right panel), of which two proteins contained signal peptides only and five proteins contained both signal peptides and transmembrane domains. Cluster4096, Cluster4499, and Cluster4500 were unique to *P. chabaudi*. Each cluster was composed of one protein with unknown function. Previous studies have revealed that ECM caused by *P. berghei* infection is mediated by the cellular immune responses [35]. Thus, the *P. chabaudi*-enriched proteins may be capable of dampening host immune responses and subsequently suppress the occurrence of ECM.

Although proteins from the same cluster are similar in their sequences, they may have differences in their immunogenicity. By exploring the RMgmDB, we identified 11 ECM-associated genes in *P. berghei.* Disruption of these genes diminishes manifestation of ECM. For example, *PBANKA_1112400* encodes an orotate phosphoribosyltransferase (OPRTase). When mice are infected with an OPRTase-deficient mutant, they do not develop symptoms of ECM. We found through sequence analysis that these 11 ECM-associated genes in *P. berghei* genome had orthologs in *P. chabaudi* genome. Gene ontology analysis revealed that two of these genes were involved in the synthesis of uracil (Supplementary Table 7), which implied that their disruption might lead to impairment of UMP synthesis. Thus, we were prompted to consider that 5-fluorouracil could be able to prevent the development of ECM. Given that ECM was mainly caused by CD8 + T-cell-mediated immune damage [36], NetMHCpan EL4.1 was adopted to predict MHC-I T-cell epitopes on ECM-related proteins and their orthologs in *P. chabaudi*. Most of the known epitopes in *P. berghei* are H2-*Db* or H2-*Kb* restricted [37]. Thus, epitope prediction was performed when the MHC alleles were H2-*Db* or H2-*Kb* gene. We found that PBANKA_1034400, PBANKA_1112400, PBANKA_0507400, and PBANKA_1401000 contained 1 to 3 T-cell epitopes when the allele was *Db*. PBANKA_1002400, PBANKA_1360100, PBANKA_1112400, PBANKA_1145800, and PBANKA_1401000 contained 1 to 3 T-cell epitopes when the MHC allele was *Kb* (Fig. 4C). Two proteins, namely, PBANKA_1349100 and PBANKA_1358000, had no predicted T-cell epitopes. They might participate in the development of ECM by an undefined mechanism. Considering that *P. chabaudi* infection does not cause cerebral malaria, the T-cell epitopes predicted in *P. chabaudi* proteins should be incapable of inducing T-cell responses. Elucidating the molecular mechanism of the occurrence of cerebral malaria induced by such *P. berghei* proteins with predicted T-cell epitopes might help develop new strategies for the prevention and therapy of cerebral malaria.

## Discussion

In this study, the network module analysis was developed and applied to discover the disease-related genes in *P. berghei* genome. Protein-coding genes shared by six *Plasmodium* species and were enriched in the rodent malaria parasites and ECM-related genes were identified. This study will improve understanding of the biological properties and pathogenesis of *P. berghei*. Through network module analysis, we identified 4127 genes shared by six *Plasmodium* species. These shared genes are involved in many aspects of biological processes, including protein phosphorylation, cell signaling, cell cycle regulation, DNA replication, gene transcription, protein transport, and protein dephosphorylation. Proteins participating in heat stress were also enriched in six *Plasmodium* species. The reason might be that *Plasmodium* needs to adapt to temperature change when transferred from mosquitoes to hosts. Most essential genes in *P. berghei* are shared by six species, which suggests that these genes are not only important for the survival of *P. berghei* but also play a critical role in the living of other *Plasmodium* species. Drugs preferentially targeting the shared genes may have a broad anti-plasmodium spectrum.

We previously reported that the enzyme for vitamin B1 synthesis was present in human malaria parasites but absent in rodent malaria parasites [38], which could partially explain why rodent malaria parasites cannot infect humans. Here, we identified genes enriched in rodent malaria parasites to further understand why *P. berghei* can parasitize mice. A total of 63 clusters consisting of 405 *P. berghei* genes were identified. Four of these clusters, namely, Cluster1, Cluster3, Cluster305, and Cluster6, were significantly enriched in rodent malaria parasites. Proteins of Cluster1 and Cluster6 contain PIR domains which are associated with chronic *Plasmodium* infection through antigenic variation. Members from Cluster3 and Cluster6 have unknown functions. However, sequence analysis indicated that several of them were exported to extracellular or integrated into the membrane of Maurer’s cleft. The remaining 59 clusters contained 68 *P. berghei* genes. Among these genes, the phenotype information of 30 genes after depletion can be obtained from RMgmDB. The phenotypic manifestation of these genes after disruption covers various stages of *Plasmodium* life cycle, especially in the hepatic stage. Some of them are essential genes and are vital for the living of *P. berghei*. Therefore, the combination of all rodent *Plasmodium* specific proteins might confer the capability of *P. berghei* of infecting mice.

The enrichment of each cluster in *P. berghei* was compared with that in *P. chabaudi* to discover ECM-related genes in *P. berghei*. Unfortunately, none of the clusters were specific to *P. berghei*. However, several clusters were enriched in *P. chabaudi*. In such situation, the occurrence of ECM caused by *P. berghei* infection might be due to two possible reasons. One is that *P. berghei* may lack certain molecules unique to or expanded in *P. chabaudi*. Cluster analysis revealed five clusters enriched in *P. chabaudi*. Although proteins in these clusters have unknown functions, sequence feature analysis showed that they are exported or membrane-associated proteins. Considering that ECM is caused by excessive host immune responses, we cannot exclude the possibility that *P. chabaudi*-enriched proteins may have the ability to suppress host immune responses. The other is that, although proteins from the same cluster are similar in sequence, their immunogenicity might be different due to amino acid variation. We collected 11 ECM-related genes from RMgmDB. Depletion of these genes results in absent of manifestation of CM. All these genes have orthologs in *P. chabaudi*. Epitope analysis revealed that some T-cell epitopes are present in several proteins of *P. berghei* but absent in the orthologs of *P. chabaudi*. Thus, the molecular mechanism of ECM mediated by CD8^+^ T-cells could be further illustrated through investigation of proteins having predicted T-cell epitopes. In addition, two ECM-related genes are involved in the synthesis of UMP. Therefore, the possible adoption of 5-Fluorouracil could be considered to reduce the occurrence of ECM.

Our study has four limitations. First, BGLL algorithm cannot be used for overlapping clusters. When a node is shared by two or more clusters, it can only be classified into one of them, which leads to inaccurate classification. The overlapping node should be split into two or more identical nodes and each of them should be classified into one of the overlapping clusters. Second, since the T cell receptors of C57BL/6 mice are restricted to *H-2 Kb* or *H-2 Db* for class I MHC, we performed T cell epitope analysis through NetMHCpan EL4.1 by setting MHC alleles to be H2-*Db* or H2-*Kb*. However, depletion of CD4 T cells was also shown to prevent the development of ECM and prolong the survival of mice infected with *P. berghei* [39, 40]. For this reason, there exists a possibility that the MHC class-I T-cell epitope negative *P. berghei* proteins might contribute to occurrence of ECM through activation of CD4 T cells. Third, the host and host-pathogen interactions should be included in the explanation of the physiopathology of ECM caused by *P. berghei*, since *P. berghei* is involved in ECM in C57BL/6 mice but not in BALB/c mice while the same parasite strain is used in both models. Finally, some of the analysis results were not confirmed by experimental investigation, but it provided value information for people working on malaria research.

## Conclusions

In conclusion, our study focused on identifying genes related to mouse diseases caused by *P. berghei* ANKA. The results showed that genes shared by all six malaria parasites are involved in various biological processes, and most of the known essential genes belong to shared genes. Genes enriched in rodent malaria parasites participate in immune escape and have a critical role in the entire *Plasmodium* life cycle, especially in the hepatic stage. Our study also highlighted that *P. berghei* ANKA lacks some proteins present in *P. chabaudi* and possesses specific T-cell epitopes, which might possibly lead to the development of cerebral malaria. Overall, our study provides new insights into the biological features and pathogenesis of *P. berghei* ANKA.

### Electronic supplementary material

Below is the link to the electronic supplementary material.

Supplementary Material 1: Table S1. The correspondence between Gene IDs and Cluster IDs.

Supplementary Material 2: Table S2. Identified *P. berghei* genes shared by six *Plasmodium* species.

Supplementary Material 3: Table S3. The enrichment of 3,367 clusters shared by six *Plasmodium* species.

Supplementary Material 4: Table S4. The enrichment of 63 clusters enriched in rodent malaria parasites.

Supplementary Material 5: Table S5. Protein features of genes enriched in 59 rodent *Plasmodium* specific clusters.

Supplementary Material 6: Table S6. The enrichment of 2 and 5 clusters enriched in *P. berghei* and *P. chabaudi*.

Supplementary Material 7: Table S7. Biological process enrichment analysis of 11 ECM-related genes derived from RMgmDB.

## Data Availability

All data were downloaded from PlasmoDB: a functional genomic database for malaria parasites. This database allows researchers to download and analyze data related to *Plasmodium* for scientific purposes. All data generated or analyzed during this study are included in this article and its supplementary information files.

## Abbreviations

*P. berghei*: *Plasmodium berghei*
*P. yoelli*: *Plasmodium yoelli*
*P. chabaudi*: *Plasmodium chabaudi*
*P. vinckei*: *Plasmodium vinckei*
ECM: Experimental cerebral malaria
DFS: Depth-first search
RMgmDB: Rodent Malaria genetically modified DataBase
PlasmoGEM: The Plasmodium Genetic Modification Project
UIS4: Sporozoites gene 4 protein
PIR: Plasmodium Interspersed Repeat
SP: Signal peptide
TM: Transmembrane domain
